# 

**Published:** 1921-03-12

**Authors:** 


### Personalia.

The Marquess of Northampton has joined the Board of Management of the Royal Hospital and Home for Incurables, Putney, of which institution the late Marquess was President for many years.

Sir Robert Hudson, G.B.E., Chairman of the Finance Committee of the Joint Committee of the British Red Cross and Order of St. John, has consented to be appointed Joint Treasurer to Westminster Hospital, in the place of the late Lord Glenconner. H.M. the King recently sanctioned the appointment of Sir Robert Hudson to be Knight of Grace of the Order of St. John of Jerusalem.

### District Medical Officers for Scotland.

The Scottish Board of Health announce the following appointments:—

Senior District Medical Officers.—Western District (Headquarters, Glasgow), R. Buchanan, M.B., C.M., D.P.H.; South-Eastern District (Headquarters, Edinburgh), A. M. McIntosh, C.M.G., M.B., Ch.B., F.R.C.S.

Junior District Medical Officers.—South-Western District (Headquarters, Glasgow), John Gilmour, M.B., C.M., F.R.C.S., D.P.H.; Northern District (Headquarters, Aberdeen), John Jeffrey, M.B., Ch.B., F.R.C.S.; Eastern District (Headquarters, Dundee), J. Murray Young, M.B., C.M.

These officers will act as medical referees in cases referred to them on questions of incapacity of insured persons for work, and as consultants in giving second opinions in questions of diagnosis and treatment. They have also a number of important administrative duties in connection with the Insurance Medical Service and other work of the Scottish Board of Health.

### "Medical Examination of Airmen."

WE have received a copy of Wing-Commander Flack's Introduction to this interesting work (John Bale, Sons & Danielsson), and understand that its omission from the copy reviewed in these columns on February 5 was due to the binder's error. This omission is, of course, now remedied.

*Erratum.*

"CAVEATS FOR DISPENSERS."—*The Hospital*, p. 494, "Formulæ in tabloid form" in line 10 to read "Formulæ in *tablet* form."

### Matrons' and Sisters' Appointments.

Royal Cornwall Infirmary, Truro.—Miss Kathleen Rowe has been appointed sister. She was trained at the Royal Albert Hospital, Devonport, where she was later sister; she has also been sister at the District Hospital, Ashton-under-Lyne.

Peel Hall Pulmonary Hospital, Preston.—Miss Leila Wood has been appointed matron. She was trained at the Preston Royal Infirmary, has been matron of the Ainsworth Pulmonary Hospital, assistant matron 2nd Western General Hospital, assistant matron the Aithen Sanatorium, Holcombe Brook, and is at present matron of the Salterley Grange Sanatorium, Birmingham.

Windsor Hospital and Convalescent Home, Felixstowe.—Miss Gertrude Fisher has been appointed sister. She was trained at the Wolverhampton General Hospital, and has held the following appointments: Ward sister at the Manchester Sanatorium; holiday sister at the Royal Sea Bathing Hospital, Margate, and at Bournemouth Sanatorium; and sister-in-charge of the Rawson Convalescent Home, Harrogate.

Isle of Ely Nursing Association.—Mrs. E. A. Kennedy-Reid has been appointed the first County Superintendent. She was trained at the Dudley Road Infirmary, Birmingham, and for several years has worked as Queen's Nurse. Latterly she has held the post of health visitor to the Isle of Ely County Council. Mrs. Reid holds the certificate of the Central Midwives Board and of the Royal Sanitary Institute for health visitors.

West End Hospital for Nervous Diseases.—Miss H. M. Goldthorp has been appointed matron. She was trained at the London Hospital, where she was subsequently sister. She has held the posts of assistant matron (holiday) of the National Hospital, Queen Square, acting matron of the Bethnal Green Military Hospital, and matron of Highfield Hospital.

Kent County Ophthalmic Hospital, Maidstone.—Miss Grace E. Coe, A.R.R.C., has been appointed lady superintendent. She was trained at the West Kent General Hospital, where she was later successively night, theatre, and x-ray sister. She has also been matron of the Hayle Place V.A.D. Hospital, Maidstone.

Colindale Hospital, Hendon.—Miss A. M. Rennie has been appointed matron. She was trained at Greenock Infirmary, afterwards becoming Queen's nurse in Midlothian, staff nurse at the Nordrach-upon-Mendip Sanatorium, sister King Edward VII. Sanatorium, Midhurst, matron of the Ochill Hills Sanatorium, Kinross-shire, and matron at Pinewood, Wokingham.

Lenzies Sanatorium, Wollsingham, Co. Durham.—Miss Martha Brown has been appointed matron. She was trained at Hull Royal Infirmary, and was later sister at the Hull and East Riding Convalescent Home, Withernsea.

Cardiff, Prince of Wales's Hospital.—Miss Maud Esther Smith has been appointed charge sister. She was trained at the North Staffordshire Infirmary, where she was later sister, and has been sister-in-charge of a private nursing home in Newcastle-under-Lyme and matron of the Red Cross Hospital, Foxlowe, Leek.

Rochford Union.—Miss Emily Howard has been appointed superintendent nurse. She was trained at Prescot Poor-law Infirmary, and has been successively night superintendent at Oldham Infirmary, home sister at Rochdale Infirmary, night superintendent at West Hartlepool Infirmary, head nurse of Lichfield Infirmary, night superintendent at Burnley Infirmary, and is at present superintendent-nurse of Wellington Infirmary.

Hertford County Hospital.—Miss C. L. Thompson has been appointed matron. She was trained at St. Bartholomew's Hospital, and holds the certificate of the Central Midwives Board. She served one year as naval sister, and has held the posts of ward sister and theatre sister at the Samaritan Hospital, and sister of medical floor and home sister at the Hampstead General Hospital.

The Infirmary, Beverley Road, Hull.—Miss Alice Johnson has been appointed midwifery sister and sister-tutor. She was trained at Bagthorpe Infirmary, Nottingham, and has been night superintendent nurse at Chesterfield Infirmary.

Royal Hospital for Sick Children, Edinburgh.—Miss Florence Blakiston has been appointed theatre sister. She was trained at the London Temperance Hospital, where she was later theatre sister, sister of men's surgical and children's wards, and night superintendent; she has also been sister-in-charge of the Royal Victoria Hospital, Netley.

Leeds Maternity Hospital.—Miss Lena A. Peacock has been appointed sister. She was trained at the Royal Halifax Infirmary and at the Maternity Nursing Association. She has engaged in private nursing in Manchester and Halifax, was night superintendent at the Alderley Edge Red Cross Hospital, and staff nurse, Q.A.I.M.N.S.R., for 4½ years, serving in France, Italy, and England.

Maternity Home, County Borough of Eastbourne.—Miss E. M. Keates has been appointed sister. She was trained at the Derbyshire Royal Infirmary and the City of London Lying-in Hospital, and has done military nursing at home and abroad.

Linacre Hospital, Bootle.—Miss S. Carr has been appointed sister. She was trained at the Union Hospital, Brownlow Hill, Liverpool, and has been sister at the Sanatorium, Stanhope, Co. Durham, and at the Helmington Row Hospital.

Royal Infirmary, Gloucester.—Miss Theo Goldsworthy has been appointed night sister. She was trained at the Royal South Hants Hospital, where she was later staff nurse; she has been sister at the Shropshire Orthopædic Hospital. Miss E. Rowe has been appointed medical ward sister and Miss E. Gardener surgical ward sister. The former was trained and was later staff nurse at the Nottingham General Hospital; the latter was trained at King Edward VII. Hospital, Windsor, has done private and district nursing at the General Hospital, Gravesend, been sister Q.A.I.M.N.S.R. at Brook Hospital, Woolwich, and done private nursing.

Clare Hall Sanatorium, South Mimms.—Miss Lilian H. Mears has been appointed sister. She was trained at Wandsworth Infirmary, has been staff nurse at the Royal Victoria Hospital, Bournemouth, has nursed under the Anglo-French Red Cross in France, when she was awarded the Medaille d'Honneur in silver, and been sister at Joyce Green Hospital, Dartford. Miss Margaret G. Hardie has also been appointed sister. She was trained at Stanley Hospital, Liverpool, has been sister at the Royal National Sanatorium, Bournemouth, and previously sister under the British Red Cross working in France and at home.

Children's Hospital, Birkenhead.—Miss Agnes M. Staley has been appointed out-patient sister. She was trained at the Worcester General Infirmary, served in France with the Anglo-French Red Cross, and in June 1920 went out with the British Committee of the Russian Red Cross to Greece.

The Infirmary, Bury.—Miss M. Ashley has been appointed matron. She was trained at Birmingham General Hospital, where she was later medical, surgical, and theatre sister; she then became successively night superintendent at Bradford Royal Infirmary, assistant matron of Halifax Royal Infirmary, and matron of the General Hospital, Darlington, and of the Saffron Walden Hospital.

Infectious Diseases Hospital, Portsmouth.—Miss Mabel Price has been appointed sister. She was trained at St. George's Infirmary, London.

Mildmay Memorial Hospital.—Miss Beatrice E. Whittingham has been appointed sister. She was trained at King's College and Queen Charlotte's Hospitals, has been night nurse at St. Luke's Hostel, on the staff of the Walthamstow Child Welfare Society, and engaged in private nursing.

